# Endogenous oestradiol and progesterone as predictors of oncogenic human papillomavirus (HPV) persistence

**DOI:** 10.1186/s12885-022-09247-3

**Published:** 2022-02-05

**Authors:** Susanne Fischer, Ulrike Kuebler, Elvira Abbruzzese, Christian Breymann, Laura Mernone, Ulrike Ehlert

**Affiliations:** 1 University of Zurich, Institute of Psychology, Clinical Psychology and Psychotherapy, Binzmuehlestrasse 14 / Box 26, 8050 Zurich, Switzerland; 2 University Hospital Zurich, Zurich, Switzerland

**Keywords:** Cervical cancer, Human papillomavirus, Oestradiol, Progesterone

## Abstract

**Background:**

High-risk human papillomavirus (HR-HPV) is the main aetiological factor for the development of cervical cancer. While nearly 70% of HR-HPV infections are cleared within 12 months, in the remainder of women they persist and can progress into cervical cancer. Oestradiol and progesterone have been shown to be involved in the development and progression of cervical cancer. The objective of this study was to investigate, for the first time, whether diurnal oestradiol and progesterone are also involved in HR-HPV persistence – before cervical cancer develops.

**Methods:**

A total of *N* = 39 women between 18 and 31 years of age were investigated. All were nulliparous and regular users of combined oral contraceptives. Presence of HR-HPV was determined by cervical swabs. Salivary oestradiol and progesterone were measured upon awakening and at 11 am, 2 pm, and 5 pm. All HR-HPV positive women were re-tested in terms of HR-HPV status 12 months later.

**Results:**

HR-HPV positive women had significantly higher morning (*p* = .007, partial eta^2^ = .221) and daily oestradiol levels (*p* < .001, partial eta^2^ = .442) when compared to HR-HPV negative women. In addition, those with persistent HR-HPV 12 months later had significantly elevated morning (*p* = .005, partial eta^2^ = .534) and daily (*p* = .027, partial eta^2^ = .346) oestradiol. Progesterone was found to be unrelated to HR-HPV.

**Conclusions:**

Oestradiol was positively linked to HR-HPV presence and persistence. Provided that these findings are replicated, regular monitoring of oestradiol levels may prove useful in identifying women who are at risk of developing cervical cancer.

## Background

Cervical cancer is the fourth most common cancer and cause of cancer death in women worldwide [1], with an incidence of over 500′000 cases per year [2]. Its development has strong aetiological links to the infection of cervical mucosa cells with high-risk human papillomavirus (HR-HPV) types, in particular HPV16 and HPV18. While around 50% of HR-HPV infections are cleared within 6 months and nearly 70% within 12 months, the infection persists in the remainder of women, in whom it can progress into cervical intra-epithelial neoplasia (CIN) and – ultimately – cervical cancer [3, 4]. Given that HR-HPV persistence is the first stage in cervical cancer development, it is vital to learn more about the conditions under which such infections persist.

One of the most promising candidate mechanisms for viral persistence is a synergistic interaction between HR-HPV infection and sex hormones, that is, oestrogens and progestogens. Indirect evidence to support this notion comes from epidemiological studies, which have shown that the intake of hormonal contraceptives and multiparity enhance the risk of developing cervical cancer in HPV-infected women [5]. As for the underlying mechanisms, it is currently assumed that upon integration of HR-HPV into the host genome, oestrogens and progestogens facilitate the expression of the oncoproteins E6 and E7 [6]. While E6 inactivates the tumour suppression protein p53, a process which results in the inhibition of apoptosis, E7 inactivates the tumour suppression protein Rb, thus stopping cell-cycle arrest. These effects are likely to be initiated via oestrogen and progesterone responsive elements within viral oncogenes. Evidence to support this hypothesis stems from in vivo experiments in mice transgenic for E6 and E7, which developed cervical cancer when oestrogens were administered [7]. In addition, clinical studies in humans have shown that progesterone levels are predictive of cervical cancer prognosis. However, as yet, no research has tested whether gonadal steroids are linked to HR-HPV persistence – *before* CIN and cervical cancer have developed.

The aim of the present study was to investigate, for the first time, whether endogenous oestradiol and progesterone concentrations predict HR-HPV persistence over the course of 12 months in otherwise healthy women. First, we hypothesized that HR-HPV positive women would exhibit higher gonadal hormone levels than HR-HPV negative women. In addition, we assumed that the higher the gonadal hormone levels at baseline, the greater the likelihood of HR-HPV persistence 12 months later.

## Methods

### Participants

This study was part of a larger study examining the influence of psychobiological factors on the course of HR-HPV (see [8] for a participant flow chart). For its main analyses, a power analyses yielded a sample size of *N* = 90 participants to detect medium-sized effects (see [8]). For the present analyses, we included all women who were regular users of combined oral contraceptives, which resulted in a total of *N* = 39 participants aged between 18 and 31 years. This sample size is similar to previous research on oestradiol/progesterone in HPV in young women [9] and was large enough to detect large-sized effects regarding the group comparisons according to a power analysis (α = .05, 1-β = .80).

All women were recruited via collaborating physicians and by various advertisements. The following exclusion criteria were applied: virginity, current or past pregnancy, acute or chronic immunological diseases (e.g., autoimmune disorders, HIV, current infectious diseases), HPV vaccination, confirmed HPV infection or cervical abnormalities in the last year, and last gynaecological check-up more than 2 years ago. At baseline, *n* = 17 women tested HR-HPV negative and *n* = 22 tested HR-HPV positive. All participants who were HR-HPV positive were re-assessed 12 months later. At follow-up, *n* = 6 women were still HR-HPV positive, while the virus had cleared in *n* = 14 participants. Two women dropped out of the study.

### Protocol

The study consisted of three parts. First, an online screening was conducted. The women were informed about the study purpose and procedures, provided informed consent, and were asked to complete questionnaires regarding sociodemographic and psychosocial information to determine eligibility.

Second, eligible participants were sent written instructions and cervical swabs to test HR-HPV status (DNAPap™ Cervical Sampler, Qiagen Gaithersburg, Inc., Maryland, USA). They were asked to take the instructions and swabs to an examination with their gynaecologist (routine check-up), which they had to schedule within 2 weeks. Participants were instructed not to use vaginal cream, lubricants or spermicides and to refrain from sexual intercourse for 3 days prior to the examination. During the examination, cervical cells were collected with the DNAPap™ Cervical Sampler. All samples were sent, on the same day, to a commercial laboratory specialised in HR-HPV screening, and analysed using the Hybrid Capture 2 High-Risk HPV DNA Test® (QIAGEN, Gaithersburg, Inc., Maryland, USA). This method allows for the detection of 13 HR-HPV types, namely HPV16, HPV18, HPV31, HPV33, HPV35, HPV39, HPV45, HPV51, HPV52, HPV56, HPV58, HPV59, and HPV68.

Third, participants were sent Salivettes® (Sarstedt, Sevelen, Switzerland) in order to collect saliva for the determination of oestradiol and progesterone. Saliva sampling had to take place within 2 weeks after the gynaecological examination and over the course of one regular workday. Measurement time points were immediately upon awakening, at 11 am, 2 pm, and 5 pm. To account for possible confounders concerning salivary hormone determination, participants were asked to strictly adhere to the following guidelines: no intense physical activity or alcoholic drinks for 24 h prior to collection of the first sample, no smoking on the day of saliva collection, and no teeth brushing, eating, or drinking for 20 min before the collection of each subsequent sample. All samples were sent to the biochemical laboratory of the Institute of Psychology at the University of Zurich, where they were stored at − 20 °C.

A follow-up investigation took place 12 months after the initial assessment. Only HR-HPV positive women were re-assessed. All women were contacted 1 month in advance in order to schedule the second gynaecological examination, where they were re-tested in terms of their HR-HPV following the same procedures as outlined above. All participants were reimbursed with 120 CHF. The study was conducted in accordance with the principles of the Declaration of Helsinki and approved by the Ethics Committee of the Faculty of Arts of the University of Zurich, Switzerland.

### Biochemical analyses

All samples were thawed, centrifuged, and analysed using commercial saliva luminescence immunoassays (IBL International GmbH, Hamburg, Germany). The inter- and intra-coefficients of variance (CVs) for oestradiol were below 14.8 and 13.3%, respectively. For progesterone, the CVs were below 18.8 and 6.0%, respectively.

### Statistical analyses

All data were tested in terms of normal distribution and homogeneity of variance. Non-normally distributed values were log-transformed. Univariate ANOVAs were conducted to compare HR-HPV negative and HR-HPV positive cases in terms of morning (awakening sample) and daily (mean of the 11 am, 2 pm, and 5 pm samples) oestradiol and progesterone. Further ANOVAs were conducted to compare cleared vs. persistent HR-HPV cases in terms of baseline oestradiol and progesterone. All analyses were controlled for age, body mass index (BMI), smoking, age at first sexual intercourse, and the number of lifetime sexual partners. The level of statistical significance was set at α = 0.05. The Statistical Package for the Social Sciences (SPSS, version 22) was used for statistical analysis.

## Results

### Descriptive statistics

Patient characteristics are presented in Table 1. The mean age of the total sample was 24 ± 3 years and the mean body mass index (BMI) was 21 ± 3 kg/m^2^. More than a third (36%) of the women were smokers. Nearly half (49%) of the women held a university degree and a further 90% had completed at least higher-track school-leaving examinations. The average age of first sexual intercourse was 17 ± 2. The average number of lifetime sexual partners was 7 ± 5. The vast majority of the women took ethinyloestradiol (0.02–0.03 mg) in combination with different progestogens (dosages between 0.075 and 3 mg). Two women took a combination of 2 mg of oestradiol valerate and 3 mg of dienogest.

**Table 1:** Participant characteristics according to the presence/absence of HR-HPV at baseline and follow-up. Medians and interquartile ranges (interquartile range in brackets), means and standard deviations (standard deviations preceded by “±”) as well as absolute and relative frequencies (relative frequencies indicated by “%”) are presented. Group comparisons between negative/positive individuals at baseline and negative/positive individuals at follow-up were conducted using Mann Whitney U tests, independent t tests, Chi-squared tests, and Fisher’s exact tests

	All participants – baseline (*n* = 39)	HR-HPV negative – baseline (*n* = 17)	HR-HPV positive – baseline (*n* = 22)	HR-HPV negative – follow-up (*n* = 14)	HR-HPV positive – follow-up (*n* = 6)
Age (years)	23 (4)	24 (4)	23 (5)	23.5 (5.8)	23 (3)
Body mass index (kg/m^2^)	20.3 (3.6)	20.8 (3.6)	19.8 (3.6)	19.7 (3.6)	19.5 (2.2)
Smoking (yes)	14 (36%)	4 (24%)	10 (46%)	7 (50%)	2 (33%)
Educational status					
Vocational training	3 (8%)	2 (12%)	1 (5%)	1 (7%)	0 (0%)
Higher school leaving exam	16 (41%)	5 (29%)	11 (50%)	5 (36%)	4 (67%)
University degree	19 (49%)	10 (59%)	9 (41%)	7 (50%)	2 (33%)
Other	1 (2%)	0 (0%)	1 (4%)	1 (7%)	0 (0%)
Age at first intercourse	16.5 ± 1.7	16.4 ± 1.4	16.6 ± 1.8	16.4 ± 2	17 ± 1.4
Number of lifetime sexual partners	6 (7)	3 (3)^a^	8.5 (10)^a^	9.5 (10)	5 (9.3)

*HR-HPV* high-risk human papillomavirus

^a^Women who were HR-HPV negative at baseline had a significantly lower number of lifetime sexual partners when compared to women who were HR-HPV positive at baseline; no other significant group differences emerged

### Gonadal hormones and HR-HPV presence

**Fig. 1:**
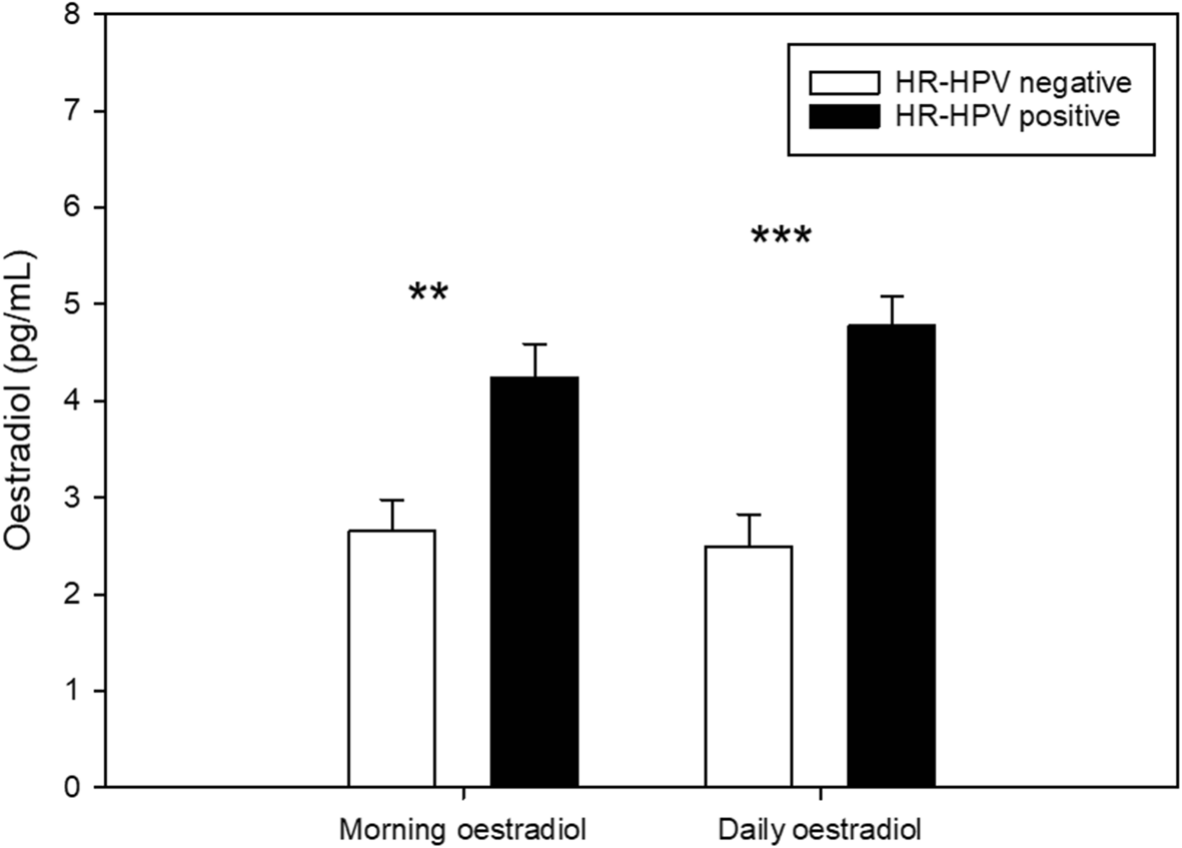
Baseline oestradiol levels in high-risk human papillomavirus (HR-HPV) negative (*n* = 17) vs. positive cases (*n* = 22). Morning oestradiol refers to the sample collected immediately upon awakening whereas daily oestradiol represents the mean of the 11 am, 2 pm, and 5 pm samples; **p < .01, ***p < .001

When controlling for age, BMI, smoking, age at first sexual intercourse and number of lifetime sexual partners, women who were HR-HPV positive had significantly higher morning (F (1, 30) = 8.49, *p* = .007, partial eta^2^ = .221) and daily oestradiol concentrations (F (1, 29) = 23.01, *p* < .001, partial eta^2^ = .442) as compared to women who were HR-HPV negative. These findings are illustrated in Fig. 1. By contrast, HR-HPV positive and negative women did not differ in morning (F (1, 30) = 0.56, *p* = .462, partial eta^2^ = .018) or daily progesterone levels (F (1, 29) = 3.03, *p* = .092, partial eta^2^ = .095).

### Gonadal hormones and HR-HPV persistence

**Fig. 2:**
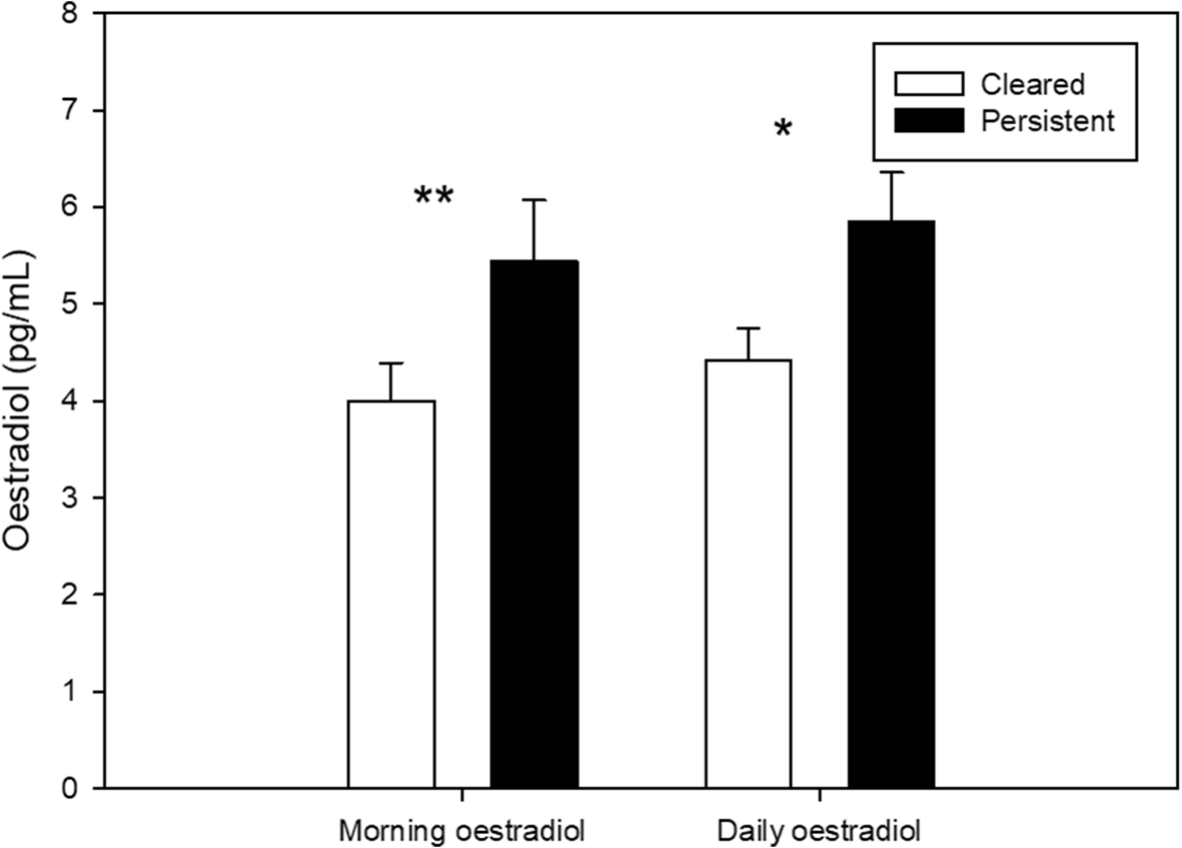
Baseline oestradiol levels in cleared (*n* = 14) vs. persistent (*n* = 6) high-risk human papillomavirus (HR-HPV) cases (12-month follow-up). Morning oestradiol refers to the sample collected immediately upon awakening whereas daily oestradiol represents the mean of the 11 am, 2 pm, and 5 pm samples; *p < .05, **p < .01

When controlling for age, BMI, smoking, age at first sexual intercourse and number of lifetime sexual partners, women with persistent HR-HPV infection at the 12-month follow-up had significantly elevated morning (F (1, 11) = 12.60, *p* = .005, partial eta^2^ = .534) and daily oestradiol levels (F (1, 12) = 6.36, *p* = .027, partial eta^2^ = .346) as compared to those with cleared HR-HPV infections. These findings are depicted in Fig. 2. By contrast, no differences between the two groups were found in terms of morning (F (1, 11) = 0.54, *p* = .478, partial eta^2^ = .047) or daily progesterone (F (1, 12) = 2.97, *p* = .111, partial eta^2^ = .198).

## Discussion

The present study yielded three findings. First, HR-HPV positive women had higher morning and daily oestradiol levels when compared to HR-HPV negative women (large effect sizes). Second, higher morning and daily oestradiol predicted the persistence of HR-HPV over the course of 12 months (large effect sizes). Third, progesterone levels were found to be unrelated to either the presence or persistence of HR-HPV infection.

The first finding is in line with epidemiological research, which has repeatedly demonstrated that both the intake of oral contraceptives and multiparity constitute risk factors for the development of cervical cancer in HR-HPV positive women [5]. The present study extends these observations by showing, for the first time, that endogenous oestradiol is associated with the presence of HR-HPV infection – before CIN and cervical cancer have developed. This stands in contrast to another study, which did not find any relationship between oestradiol and HR-HPV presence [10]. However, this study only looked at participants with abnormally low or high oestradiol levels, thus failing to examine any potential associations between oestradiol and HR-HPV as they occur in the normal endocrine range. In accordance with our findings, research in younger women has shown that high-oestradiol states, such as during the ovulatory phase of the menstrual cycle [9] and the pre- versus post-partum period [11], are linked to higher levels of HR-HPV. Moreover, one study provided evidence for a positive association between oestrogen receptor transcripts at the cervix and HR-HPV infection [12]. One explanation for these findings may be that oestrogens impact the proliferation and maturation of cervical epithelial cells and/or viral replication, resulting in a greater number of viral DNA copies and thus enhancing the probability of a positive cervical swab test result.

The second finding complements the first finding by providing initial evidence that oestradiol may predict HR-HPV persistence. As a potential explanation, oestradiol may compromise innate and acquired immune responses, thus contributing to the maintenance of high levels of HR-HPV. Indeed, a seminal study in healthy women demonstrated that in vitro stimulation with HPV16 virus-like particles combined with an exogenous administration of oestradiol and progesterone decreased lympho-proliferation and pro-inflammatory cytokine production while increasing anti-inflammatory cytokines and transcriptional regulators of regulatory T cells [13]. This notion echoes the results of a previous report by our group, which found that chronic stress and the stress hormone cortisol, an important modulator of immune responses, were linked to HR-HPV status at baseline and follow-up [8].

The third finding, which is of a lack of any association between progesterone and HR-HPV infection and persistence thereof, is in line with a previous study in middle-aged women, which did not find women with abnormally low or high progesterone levels to have a higher risk of concomitant HR-HPV infection [10]. Notably, epidemiological research has not provided convincing evidence that progestogenic contraceptives enhance the risk of cervical cancer, whereas animal studies and studies on human cell lines are supportive of its role as a co-factor in cervical cancer development and progression [7]. In sum, the jury is still out on whether progesterone plays a role in cervical cancer, and further research in healthy women is necessary to illuminate its role in HR-HPV persistence.

The present study encompasses several strengths. First, to the best of our knowledge, it is the first prospective examination of sex hormones and HR-HPV persistence. Second, we investigated a homogenous sample of healthy, nulliparous young women, and were careful to eliminate or control for any potential confounders of our results (e.g., other ongoing infections, smoking). Third, we employed a repeated sampling schedule to assess sex hormones, thus enhancing the reliability of our findings. However, a number of limitations are equally worthy of mention. First, this was a pilot study and our sample size was small, which limits the representativeness of our findings. Furthermore, this study was likely underpowered to detect any significant effects regarding progesterone. An independent replication of our findings is thus warranted, preferably in a large, more representative cohort of women. Second, and related to this, as our study was confined to young women on combined oral contraceptives, the present findings cannot be generalised to naturally cycling and post-menopausal women. Finally, we were unable to differentiate between different subtypes of HR-HPV, which may interact differently with sex hormones.

When taken together, these findings suggest that higher levels of oestradiol may already be detrimental at the time of HR-HPV infection − before CIN and cervical cancer have developed. These findings may be relevant to the prevention of HR-HPV infection, insofar as the monitoring of endogenous oestradiol levels and the choice of appropriate contraception could help to enhance vaccine efficacy. However, further research on the role of sex hormones in the acquisition and persistence of HR-HPV in different age groups is necessary in order to develop strategies that are tailored to the needs of the individual woman.

## Data Availability

The ethical approval for this study dates back to 2012 and does not allow us to share the patient data on a repository. However, the data set of the present study is available from the corresponding author on reasonable request.

## Abbreviations

BMI: Body mass index
CIN: Cervical intra-epithelial neoplasia
HR-HPV: High-risk human papillomavirus
CV: Coefficients of variance

## References

[1] GLOBOCAN (2012). Estimated cancer indicidence, mortality and prevalence worldwide in 2012.

[2] GLOBOCAN (2018). Estimated cancer indicidence, mortality and prevalence worldwide in 2018.

[3] Schiffman M, Castle PE, Jeronimo J, Rodriguez AC, Wacholder S (2007). Human papillomavirus and cervical cancer. Lancet.

[4] de Sanjose S, Brotons M, Pavon MA (2018). The natural history of human papillomavirus infection. Best Pract Res Clin Obstet Gynaecol.

[5] Moodley M, Moodley J, Chetty R, Herrington CS (2003). The role of steroid contraceptive hormones in the pathogenesis of invasive cervical cancer: a review. Int J Gynecol Cancer.

[6] Ramachandran B (2017). Functional association of oestrogen receptors with HPV infection in cervical carcinogenesis. Endocr Relat Cancer.

[7] Hellberg D (2012). Sex steroids and cervical cancer. Anticancer Res.

[8] Kuebler U, Fischer S, Mernone L, Breymann C, Abbruzzese E, Ehlert U (2021). Is stress related to the presence and persistence of oncogenic human papillomavirus infection in young women?. BMC Cancer.

[9] Liu SH, Brotman RM, Zenilman JM, Gravitt PE, Cummings DA (2013). Menstrual cycle and detectable human papillomavirus in reproductive-age women: a time series study. J Infect Dis.

[10] Kedzia W, Gozdzicka-Jozefiak A, Kwasniewska A, Schmidt M, Miturski R, Spaczynski M (2000). Relationship between HPV infection of the cervix and blood serum levels of steroid hormones among pre- and postmenopausal women. Eur J Gynaecol Oncol.

[11] Arena S, Marconi M, Ubertosi M, Frega A, Arena G, Villani C (2002). HPV and pregnancy: diagnostic methods, transmission and evolution. Minerva Ginecol.

[12] Shew ML, McGlennen R, Zaidi N, Westerheim M, Ireland M, Anderson S (2002). Oestrogen receptor transcripts associated with cervical human papillomavirus infection. Sex Transm Infect.

[13] Marks MA, Gravitt PE, Burk RD, Studentsov Y, Farzadegan H, Klein SL (2010). Progesterone and 17beta-estradiol enhance regulatory responses to human papillomavirus type 16 virus-like particles in peripheral blood mononuclear cells from healthy women. Clin Vaccine Immunol.

